# Supplementary material for: Canine EEG helps human: cross-species and cross-modality epileptic seizure detection via multi-space alignment
Source: Natl Sci Rev. 2025 Mar 4;12(6):nwaf086. doi: 10.1093/nsr/nwaf086 (PMC12051906; doi:10.1093/nsr/nwaf086)
Supplement: nwaf086_Supplemental_File [file nwaf086_supplemental_file.pdf]

SUPPLEMENTARY INFORMATION

S1: The proposed MSA

Input space normalization Inter-species variability leads to significant discrepancies in the marginal probability distributions of EEG signals. Euclidean Alignment (EA) [1] has been demonstrated to be effective in EEG-based classification tasks like motor imagery. This unsupervised approach aligns EEG data from different patients within the same species and across species, making subsequent analyses easier and more robust.

We apply EA to each subject separately to mitigate individual differences, and then combine all aligned EEG trials from the source subjects into a single source domain.

For a subject with N EEG trials $\{X^n\}_{n=1}^N$, EA first computes their mean covariance matrix:

$$\bar{R} = \frac{1}{N}\sum_{n=1}^{N} X^n \left(X^n\right)^\top , \tag{1}$$

and then performs the alignment by

$$\tilde{X}^n = \bar{R}^{-1/2} X^n . \tag{2}$$

$\tilde{X}^n$ then replaces X^n in all subsequent operations.

This process is repeated for each subject in the source and target species to normalize its mean covariance matrix to the identity matrix, making them more consistent.

Input space alignment As illustrated in Fig. S1b, addressing the disparity in channel dimensionality is pivotal to effective cross-species transfer. Our proposed solution combines a Transformer Encoder with a linear layer in a neural network architecture, referred to as ResizeNet.

The Transformer Encoder captures the intricate temporal dependencies and spatial relationships in the EEG data, reflecting the inter-connections between distinct channel locations using a self-attention mechanism. Subsequently, a linear layer is employed to project EEG data from a higher dimensionality to a lower space, ensuring consistent input to the subsequent feature extractor.

Let the original EEG data be represented as $X \in \mathbb{R}^{N \times C \times T_s}$, where N is the training batch size, C the number of channels, and Ts the number of time samples. The EEG data are first reshaped to $X \in \mathbb{R}^{N \times T_s \times C}$, and then input to the Transformer Encoder, which includes multi-head self-attention and a feedforward neural network. After this, a linear layer reduces the dimensionality for species with more channels. For instance, the human sEEG data in the CHSZ dataset have 18 channels, whereas the canine iEEG data in the Kaggle dataset have 16 channels; when transferring from the CHSZ dataset to the Kaggle dataset, ResizeNet reduces the human sEEG data to 16 channels to match the dimensionality of the canine iEEG data.

The final ResizeNet transformation is expressed as:

$$\hat{X} = R(T(R(X)) \cdot W_L), \tag{3}$$

where $R(\cdot)$ is the reshape function used for switching the last two dimensions of data, $T(\cdot)$ the Transformer Encoder with two layers and two heads, and $W_L \in \mathbb{R}^{C \times C_t}$ the learned parameter matrix, with C_t being the target number of channels.

Feature space alignment After ResizeNet alignment that uniforms the input signal dimensionality from both source and target species, domain adaptation can then be conducted to further align the feature distributions.

Domain adaptation leverages data from the labeled source domain $\mathcal{D}_s = \{(\mathbf{X}_s^i, y_s^i)\}_{i=1}^{N_s}$ and the unlabeled target domain $\mathcal{D}_t = \{(\mathbf{X}_t^i)\}_{i=1}^{N_t}$ to minimize their discrepancies, enabling the model trained on the source domain to generalize to the target domain. Traditional domain adaptation methods mainly consider the simpler homogeneous domain adaptation, i.e., the feature spaces of the source and target domains are identical ($\mathcal{X}_s = \mathcal{X}_t$), but their probability distributions are different ($P(X_s, y_s) \neq P(X_t, y_t)$).

The loss function of domain adaptation using MMD is:

$$
\begin{aligned}
L_{\mathrm{DA}} &= \mathrm{MMD}^2\left(X_s, \hat{X}_t\right) \\
&= \left\| \frac{1}{N_s} \sum_{i=1}^{N_s} \phi\left(\mathrm{x}_i^s\right) - \frac{1}{N_t} \sum_{j=1}^{N_t} \phi\left(\hat{\mathrm{x}}_j^t\right) \right\|_{\mathcal{H}}^2,
\end{aligned} \tag{4}
$$

where $\mathcal{H}$ is a reproducing kernel Hilbert space with a feature mapping ϕ, and $\hat{X}_t$ is the ResizeNet transformation of X_t defined in Eq. (3). Extensions of MMD, e.g., joint MMD [2] and local MMD [3], can also be utilized in domain adaptation regularization.

Output space alignment We utilizes knowledge distillation to further align the output spaces. Knowledge distillation forces the student model to mimic the teacher model's behavior by imposing a stringent congruent constraint on their predictions, typically utilizing the Kullback–Leibler divergence.

As illustrated in Fig. S1c, the original EEG signals with C channels are first reduced to C_t channels by ResizeNet, or through simple channel selection (the first C_t channels were used in this paper). In this way, we obtain two different signals with the same shape, one by ResizeNet and the other by channel selection. A neural network is then used to compute z_r, the logits from ResizeNet, and z_s, the logits from channel selection. Subsequently, knowledge distillation is performed on z_r and z_s:

$$
L_{\mathrm{KD}} = \frac{1}{N} \sum_{i=1}^{N} \tau^2 KL\left(\boldsymbol{p}_r, \boldsymbol{p}_s\right), \tag{5}
$$

where τ is a relaxation parameter (referred to as the temperature in [4]) to soften the output of the teacher network, and $p = \frac{\exp(z_k/\tau)}{\sum_j \exp(z_j/\tau)}$ is the softmax operation, with z_i being the logit for the i-th class and n the total number of classes.

Multi-space alignment To minimize the gaps between species, we perform the above input-space, feature-space, and output-space alignments simultaneously. In the proposed cross-species transfer learning framework shown in Fig. S1a, we first perform EA and ResizeNet to match the input dimensionality of the source and target species. Then, domain adaptation is performed to further reduce the discrepancies in the feature space. Finally, knowledge distillation is performed on the logits from ResizeNet and channel selection strategy.

The overall optimization objective is composed of three parts: the supervised cross-entropy loss, the domain adaptation loss, and the knowledge distillation loss:

$$
L = L_{\mathrm{CE}} + \lambda \cdot L_{\mathrm{KD}} + \beta \cdot L_{\mathrm{DA}}, \tag{6}
$$

where L_{CE} is the classic cross-entropy loss for supervised learning, and λ and β are trade-off hyperparameters.

To summarize, EA normalizes the input data to a unified scale, ResizeNet reduces higher-dimensional data to a lower-dimensional representation, domain adaptation aligns feature distributions, and knowledge distillation ensures consistency in model predictions. Together, these alignments form a unified framework tailored to address the complexities of cross-species and cross-modality EEG analysis. By integrating innovations across the input, feature, and output spaces, the proposed approach achieves effective data normalization, robust feature representation, and consistent predictive performance.

The proposed MSA framework is illustrated in Fig. S1. ResizeNet (Fig. S1b) incorporates a Transformer encoder, a linear layer, and two reshape modules. MSA (Fig. S1a) integrates alignments across input, feature, and output spaces for cross-species adaptation. Specifically, ResizeNet addresses input space discrepancies, domain adaptation aligns cross-species data in the feature space (Fig. S1d), and knowledge distillation ensures output space alignment between species (Fig. S1c).

S2: Algorithms under comparison

Details of the training and test set splits for the three experiment scenarios (within-species, unsupervised cross-species transfer, and semi-supervised cross-species transfer) are illustrated in Fig. S2.

Five popular unsupervised domain adaptation approaches were compared:

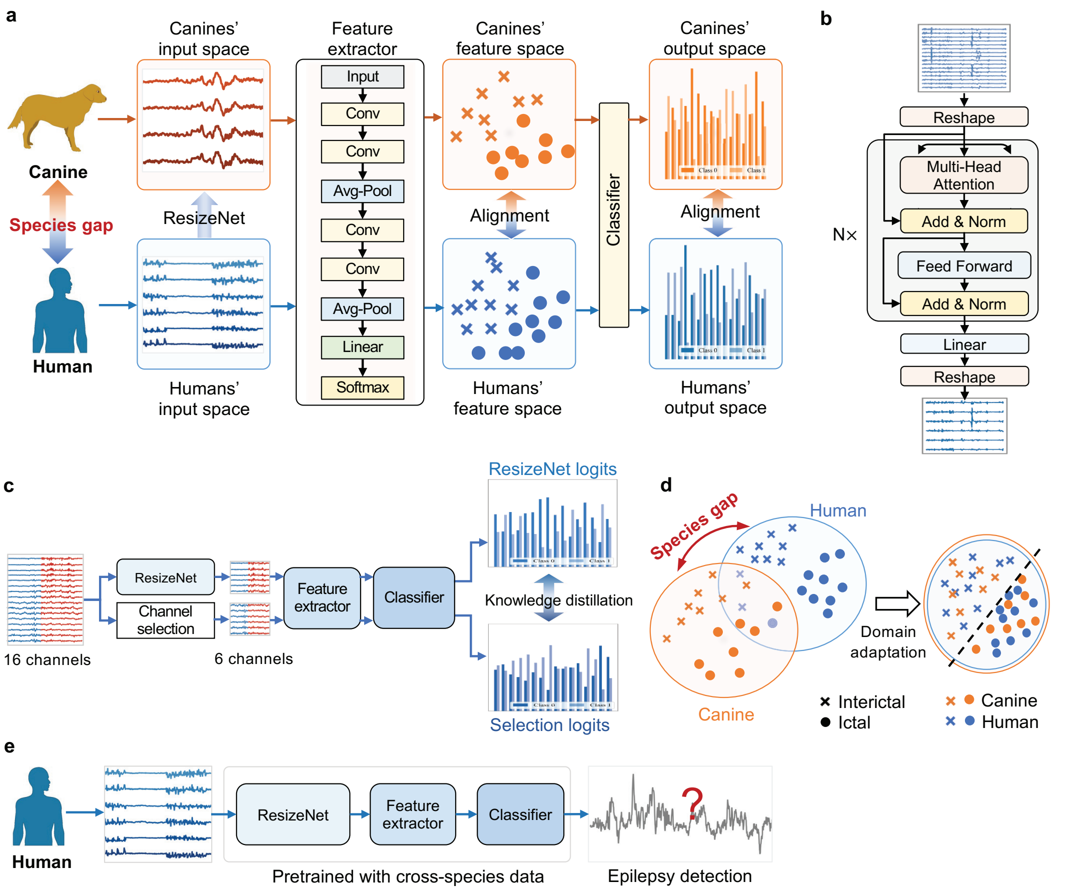

Figure S1. Overview of the proposed cross-species and cross-modality epilepsy seizure detection framework. (a) Training of the cross-species and cross-modality transfer network utilizes iEEG/sEEG data from both canines and humans. (b) The proposed ResizeNet, which projects EEG signal of the species with higher dimensionality (collected with more EEG electrodes) to a lower dimensionality to match their feature spaces. (c) The integration of knowledge distillation with ResizeNet for output-space alignment. (d) Domain adaptation for distribution matching to achieve feature-space alignment. (e) Illustration of the test phase on human iEEG/sEEG data.

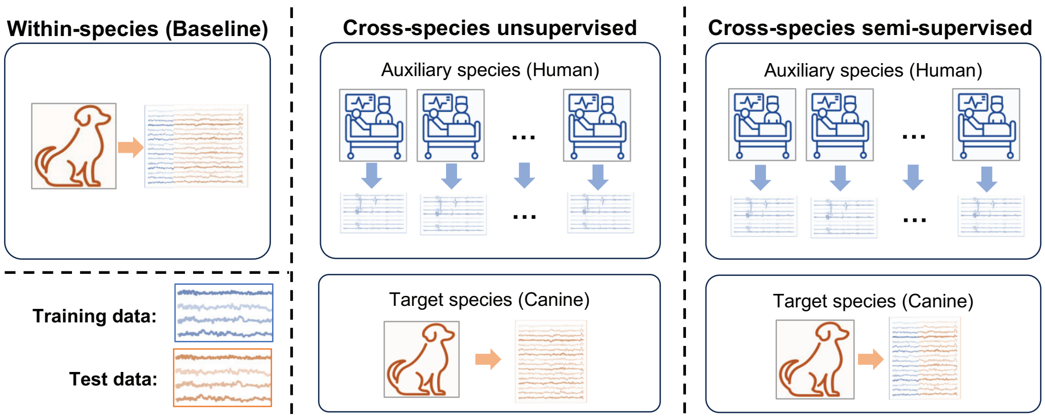

Figure S2. Illustration of three experiment scenarios, taking the Human-to-Canine transfer task as an example.

1. Deep Adaptation Network (DAN) [5], which achieves feature alignment by minimizing the maximum mean discrepancies (MMD) [6] in the feature space.
2. Joint Adaptation Network (JAN) [2] and Deep Subdomain Adaptation Network (DSAN) [3], which construct transfer networks by aligning class-conditioned subdomain distributions based on local MMD in the feature space, utilizing either model prediction probabilities or pseudo-labels.
3. Minimum class confusion (MCC) [7], which integrates a weighted prediction entropy with class correlation to reduce class confusion in the output space.
4. Source hypothesis transfer (SHOT) [8], which utilizes information maximization for conditional entropy minimization and label marginal entropy regularization in the output space.

Six popular and diverse knowledge distillation approaches were combined with the proposed ResizeNet to further improve the alignment performance:

1. Attention Transfer (AT) [9], which utilizes an attention mechanism to enhance the student network's performance by mapping attention from the teacher's feature maps.
2. Neuron Selectivity Transfer (NST) [10], which treats knowledge transfer as a distribution matching problem by aligning neuron selectivity patterns between networks.
3. Similarity-Preserving (SP) [11], which ensures the preservation of pairwise similarities between activations in the teacher network to maintain relational integrity in the student network.
4. Relational Knowledge Distillation (RKD) [12], which focuses on transferring mutual relations of data samples using distance-wise and angle-wise distillation losses.
5. Probabilistic Knowledge Transfer (PKT) [13], which aligns the probability distributions in the feature space, rather than directly mapping the features.
6. Correlation Congruence (CC) [14], which transfers correlations between instances using a generalized kernel method based on the Taylor series expansion.

The classification accuracies (%) of the baseline methods and the proposed ResizeNet+ approaches are summarized in Table S1.

Table S1. Average classification accuracies (%) of unsupervised and semi-supervised cross-species transfer on Kaggle dataset. The best average accuracy of each task is marked in bold, and the second best by an underline.

Approach		Canine-to-Human						Human-to-Canine					
		0%	5%	10%	15%	20%	Avg.	0%	5%	10%	15%	20%	Avg.
Baseline	Within	/	81.77	83.13	86.31	86.72	84.48	/	85.33	87.61	90.39	91.73	88.77
	Comb.	79.21	88.52	89.17	89.20	89.86	87.19	88.21	90.31	90.01	90.29	89.76	89.72
	DAN	85.11***	87.14	88.58	89.29	90.41	88.11	85.25	90.48	91.46*	92.90**	94.17**	90.85
	JAN	85.03****	86.32	87.33	88.40	89.66	87.35	85.11	89.41	91.17*	93.10***	93.22*	90.40
	SHOT	72.74	77.00	78.92	80.28	81.90	78.17	75.60	80.90	84.84	85.79	86.54	82.73
	DSAN	82.89*	86.27	86.91	88.66	87.52	86.45	86.45	90.58	92.34**	93.30	94.16**	91.37
	MCC	75.89	87.34	89.54	89.75	91.59	86.82	80.08	92.03	92.11**	93.84***	93.85**	90.38
ResizeNet+	AT	82.92	89.56*	90.88**	92.14*	93.58**	89.82	89.28*	90.59	91.08	92.68*	93.66**	91.46
	NST	85.02***	87.49	88.23	89.01	89.70	87.89	88.35	91.39	91.66	91.38	91.40*	90.84
	SP	84.38**	89.32	**91.27***	92.24	94.06**	90.25	89.10	90.75	91.91**	92.59*	92.89*	91.45
	RKD	80.22	89.14	91.07**	91.20	92.65**	88.86	88.04	90.96	91.94	91.37	92.93	91.05
	PKT	83.04*	89.54	90.41	91.77	**93.65****	89.68	89.18	90.75	92.21**	92.66*	93.77**	91.71
	CC	85.19***	89.84	91.53*	92.24*	93.46*	90.45	88.35	91.07	91.30	91.83	92.68*	91.05
	MSA	**85.55*****	**90.29****	91.26**	**92.34***	93.54**	**90.60**	**89.58***	**92.90***	**93.72*****	**94.80*****	**95.05*****	**93.21**

****: $p < 0.0001$; ***: $p < 0.001$; **: $p < 0.01$; *: $p < 0.05$.

S3: Dataset information

The Kaggle dataset includes data from eight human patients and four canines, provided by the Mayo Clinic and University of Pennsylvania. The canines were continuously monitored via video and iEEG. The iEEG data were acquired from an implanted device with a sampling rate of 400 Hz, utilizing 16 subdural electrodes arranged on two standard, human-sized, 4-contact strips implanted in an anteroposterior position on each hemisphere. The remaining eight human patients had drug-resistant epilepsy and were undergoing iEEG monitoring at Mayo Clinic Rochester. These signals

were continuously sampled at either 500 or 5000 Hz, using varying subdural electrode grids based on individual clinical considerations [15].

The Freiburg dataset comprises invasive long-term iEEG recordings from 21 human patients, acquired at a sampling rate of 256 Hz during invasive pre-surgical epilepsy monitoring at the Epilepsy Center of the University Hospital of Freiburg, Germany. The iEEG signals were recorded with three focal and three extra-focal electrode contacts [16].

The CHSZ dataset consists of sEEG recordings from 27 children, aged three months to ten years. The original sampling rate was either 500 Hz or 1000 Hz. Each subject had one to six seizure events. Experts annotated the onset and offset of each seizure for every child [17].

The NICU dataset documented neonatal seizures from 79 full-term neonates, with a sampling frequency of 256 Hz and a median recording duration of 74 minutes (interquartile range: 64 to 96 minutes). Three experts independently annotated each second of sEEG data, yielding an average of 460 seizures per expert. According to their consensus, 39 neonates experienced seizures, and these data were incorporated into our experiments [18].

Table S2 shows the main characteristics of four datasets. Tables S3-S5 provide detailed characteristics for each subject in the Kaggle, Freiburg and CHSZ datasets, respectively.

Table S2. Summary of the four epilepsy datasets.

Dataset	EEG Type	# Patients	# Channels	Sampling rate (Hz)	Signal length (second)	# Ictal trials	# Interictal trials
Kaggle	Intracranial	4 canines	16	400	1	1,087	9,116
		8 humans	[16, 72]	500 or 5000	1	1,390	14,329
Freiburg	Intracranial	21 humans	6	256	1	21,000	189,000
NICU	Scalp	39 humans	18	256	4	11,912	40,622
CHSZ	Scalp	27 humans	18	500	4	716	20,521

Table S3. Characteristics of the four canine subjects and eight human subjects in the Kaggle dataset.

Species	ID	# Channels	Sampling rate (Hz)	# Seizures	# Ictal trials	# Interictal trials	# Total trials
Canine	1	16	400	9	178	418	596
	2	16	400	5	172	1,148	1,320
	3	16	400	22	480	4,760	5,240
	4	16	400	6	257	2,790	3,047
Human	1	68	500	7	70	104	174
	2	16	5,000	7	151	2,990	3,141
	3	55	5,000	9	327	714	1,041
	4	72	5,000	5	20	190	210
	5	64	5,000	7	135	2,610	2,745
	6	30	5,000	8	225	2,772	2,997
	7	36	5,000	6	282	3,239	3,521
	8	16	5,000	4	180	1,710	1,890

S4: Data preprocessing

For the NICU and CHSZ datasets, the original sEEG signals were acquired using 19 unipolar electrodes positioned according to the international 10–20 system, from which 18 bipolar channels were derived [17]: Fp2-F4, F4-C4, C4-P4, P4-O2, Fp1-F3, F3-C3, C3-P3, P3-O1, Fp2-F8, F8-T4, T4-T6, T6-O2, Fp1-F7, F7-T3, T3-T5, T5-O1, Fz-Cz, and Cz-Pz.

The EEG signals in all four datasets entered a 50 Hz notch filter and a 0.5-50 Hz bandpass filter, and were then segmented into 1-second non-overlapping trials in the Kaggle and Freiburg datasets, and 4-second trials in the CHSZ and NICU datasets, in line with [17].

In the NICU dataset, EEG segments were independently annotated by three experts on a per-second basis, with groundtruth labels determined by majority consensus. In the other three datasets, a single expert annotated the beginning and end of each seizure. For each cross-species transfer task, the duration of time segments was unified across the source and target species. For instance, when transferring from canines to humans in the Kaggle dataset, the human iEEG sig-

Table S4. Characteristics of the 21 human subjects in the Freiburg dataset.

ID	Sex	Age	H/NC[1]	Origin of seizure	# Seizures	# Ictal trials	# Interictal trials	# Total trials
1	f	15	NC	Frontal	4	25,200	86,400	111,600
2	m	38	H	Temporal	3	20,243	86,400	106,643
3	m	14	NC	Frontal	5	29,480	86,400	115,880
4	f	26	H	Temporal	5	36,000	86,400	122,400
5	f	16	NC	Frontal	5	35,630	86,400	122,030
6	f	31	H	Temporo/Occipital	3	23,100	86,400	109,500
7	f	42	H	Temporal	3	21,600	88,597	110,197
8	f	32	NC	Frontal	2	12,871	86,979	99,850
9	m	44	NC	Temporo/Occipital	5	36,000	86,163	122,163
10	m	47	H	Temporal	5	38,535	88,047	126,582
11	f	10	NC	Parietal	4	28,800	86,570	115,370
12	f	42	H	Temporal	4	28,800	178,652	207,452
13	f	22	H	Temporo/Occipital	2	14,400	86,400	100,800
14	f	41	H, NC	Fronto/Temporal	4	25,200	85,894	111,094
15	m	31	H, NC	Temporal	4	36,000	86,400	122,400
16	f	50	H	Temporal	5	42,075	86,400	128,475
17	m	28	NC	Temporal	5	53,285	86,634	139,919
18	f	25	NC	Frontal	5	46,698	89,569	136,267
19	f	28	NC	Frontal	4	46,800	87,780	134,580
20	m	33	NC	Temporo/Parietal	5	46,472	92,219	138,691
21	m	13	NC	Temporal	5	43,200	86,177	129,377

[1] H: Hippocampal origin; NC: Neocortical origin.

Table S5. Characteristics of the 27 subjects in the CHSZ dataset.

ID	Age	Seizure subtype[1]	# Seizures	# Ictal trials	# Interictal trials	# Total trials
1	10m	Tonic-Clonic	1	21	170	191
2	7m	Tonic-Clonic	1	13	28	41
3	3m	Tonic-Clonic	1	21	100	121
4	2y1m	Tonic-Clonic	3	11	51	62
5	8y11m	Partial	1	46	16	62
6	4y7m	Tonic-Clonic	1	11	133	144
7	10y10m	Absence	1	10	16	26
8	1y8m	Tonic-Clonic	1	24	13	37
9	1y7m	Tonic-Clonic	1	14	52	66
10	1y8m	Partial	1	50	3,549	3,599
11	1y6m	Partial	4	143	2,595	2,738
12	2y11m	Partial	1	16	3,583	3,599
13	3m	Partial	1	30	224	254
14	3y7m	Absence	6	9	2,560	2,569
15	6y4m	Absence	6	27	158	185
16	3m	Partial	1	33	9	42
17	2y8m	Tonic-Clonic	1	44	53	97
18	3y11m	Absence	3	12	600	612
19	3m	Partial	1	16	1,321	1,337
20	6y3m	Absence	6	16	1,031	1,047
21	6m	Partial	1	23	82	105
22	8y10m	Tonic-Clonic	2	30	36	66
23	8y6m	Partial	4	8	77	85
24	3y10m	Tonic-Clonic	2	33	64	97
25	4y6m	Tonic-Clonic	2	29	89	118
26	7y8m	Partial	1	10	328	338
27	5m	Partial	1	16	3,583	3,599

[1] An expert annotated the beginning and end of each seizure and its subtype (tonic-clonic seizure, absence seizure, or partial seizure) for each child.

nals were downsampled to 400 Hz using the resample function in the MNE package (`https://mne.tools/stable/index.html`).

S5: Cross-dataset human-to-human transfer experiments

Within-species cross-dataset experiments were conducted to validate the effectiveness of the proposed ResizeNet+MSA approach.

There were significant between-dataset discrepancies in this setting. For example, the CHSZ dataset includes 18 sEEG channels sampled at 400 Hz, whereas the Kaggle-Human dataset consists of 16-72 iEEG channels sampled at 500 Hz. Although these datasets originate from the same species, they differ significantly in acquisition methods, channel numbers and locations, and sampling rates. Based on these variations, we explored three experiment settings, as illustrated in Fig. S3:

1. Cross-dataset, within-species (human-to-human) and within-modal transfer: In this scenario, models were trained on human sEEG data from the NICU or CHSZ dataset and tested on another human sEEG dataset. Since there were no channel differences between the NICU and CHSZ datasets, the proposed ResizeNet+MSA approach was not applied in this setting.
2. Cross-dataset, within-species (human-to-human) and cross-modal transfer: Models were trained on human iEEG or sEEG datasets and tested on human datasets of a different modality (e.g., iEEG to sEEG, or sEEG to iEEG). Input discrepancies exist in this setting, making alignment necessary.
3. Cross-dataset, multi-species [(canine+human)-to-human] and multi-modal transfer: Canine iEEG and human iEEG/sEEG datasets were combined as the training set, with CHSZ used as the test dataset. This setting involves both multi-species and multi-modal data, introducing significant input discrepancies.

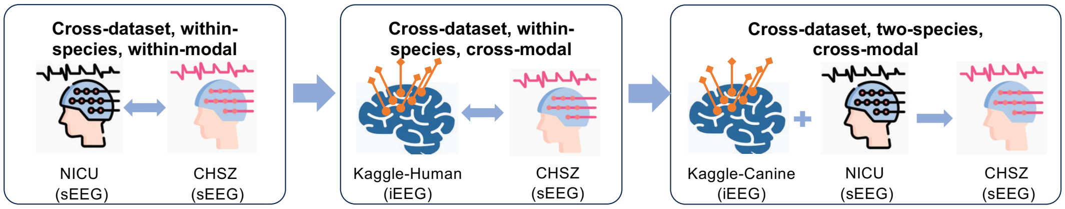

Figure S3. Illustration of three cross-dataset transfer scenarios.

Table S6 shows the experiment results:

1. The cross-dataset within-species transfer performance was better than basic cross-species transfer without additional techniques.
2. ResizeNet+MSA demonstrated its effectiveness in cross-dataset cross-modal experiments.
3. Combining multi-species data in the training set and testing on a human dataset resulted in the best performance.
4. For the within-modal scenario, NICU and CHSZ datasets have the same number and placement of channels, so there were no data heterogeneous discrepancies. UDA approaches were not always effective. Compared to the cross-modal scenario, within-modal transfer achieved better performance, e.g., 85.17% > 84.37% and 68.87% > 67.24%, indicating that cross-modal transfer (with data heterogeneous discrepancy) is indeed more challenging.
5. For the cross-modal scenario, iEEG and sEEG signals, collected from different devices, introduce significant data heterogeneity. While UDA approaches may be effective in some cases, the proposed ResizeNet framework consistently outperformed others. Notably, ResizeNet+MSA achieved the highest performance among all approaches.
6. For the multi-modal scenario, we further combined two-species datasets to build the training set. With the increasing number of training data, the performance improved, e.g., 84.37% < 86.33% < 87.05%. With the help of ResizeNet+MSA, the best performance was achieved on the CHSZ dataset, e.g., 91.34%, surpassing models trained on single-species single-modal data. This improvement likely stemmed from the increased training data volume and the proposed MSA, which effectively handles significant data heterogeneities.

Table S6. Average unsupervised cross-dataset transfer AUCs (%) on NICU and CHSZ datasets. The best average performance of each task is marked in bold, and the second best by an underline.

Approach		Within-species within-modal[1]		Within-species cross-modal[2]		Multi-species multi-modal[3]	
		NICU -to- CHSZ (sEEG -to- sEEG)	CHSZ -to- NICU (sEEG -to- sEEG)	Kaggle (H) -to- CHSZ (iEEG -to- sEEG)	CHSZ -to- Kaggle (H) (sEEG -to- iEEG)	NICU+Kaggle (C) -to- CHSZ (iEEG+sEEG -to- sEEG)	NICU+Kaggle (C+H) -to- CHSZ (iEEG+sEEG -to- sEEG)
Baseline[4]	Source Only[6]	**85.17**$_{\pm1.3}$	68.87$_{\pm1.0}$	84.37$_{\pm0.1}$	67.24$_{\pm1.3}$	86.33$_{\pm0.69}$	87.05$_{\pm1.04}$
	DAN	82.28$_{\pm2.1}$	68.84$_{\pm0.1}$	76.75$_{\pm2.8}$	72.66$_{\pm3.5}$	82.57$_{\pm1.13}$	82.72$_{\pm1.69}$
	JAN	80.81$_{\pm0.9}$	64.52$_{\pm0.7}$	70.01$_{\pm1.7}$	$\underline{73.79}_{\pm1.9}$	81.35$_{\pm1.86}$	80.10$_{\pm3.34}$
	SHOT	80.03$_{\pm1.1}$	68.82$_{\pm0.2}$	72.75$_{\pm3.5}$	53.92$_{\pm1.5}$	74.44$_{\pm4.08}$	80.86$_{\pm1.00}$
	DSAN	68.97$_{\pm2.5}$	**69.66**$_{\pm0.1}$	67.99$_{\pm1.8}$	66.51$_{\pm3.6}$	60.09$_{\pm1.14}$	60.90$_{\pm1.21}$
	MCC	$\underline{83.71}_{\pm0.3}$	$\underline{69.62}_{\pm0.2}$	78.11$_{\pm4.3}$	56.05$_{\pm1.8}$	75.27$_{\pm4.62}$	76.60$_{\pm5.40}$
ResizeNet+[5]	AT	-	-	84.29$_{\pm1.4}$	67.03$_{\pm5.3}$	89.14$_{\pm1.09}$	88.94$_{\pm0.77}$
	NST	-	-	$\underline{84.87}_{\pm1.7}$	70.11$_{\pm1.0}$	89.07$_{\pm0.15}$	$\underline{89.46}_{\pm0.28}$
	SP	-	-	84.06$_{\pm1.1}$	67.05$_{\pm5.1}$	89.19$_{\pm0.60}$	89.26$_{\pm0.40}$
	RKD	-	-	80.40$_{\pm1.9}$	66.76$_{\pm0.9}$	88.48$_{\pm0.55}$	89.21$_{\pm0.08}$
	PKT	-	-	84.14$_{\pm1.1}$	67.11$_{\pm5.2}$	88.84$_{\pm0.40}$	88.87$_{\pm0.46}$
	CC	-	-	83.54$_{\pm1.2}$	69.26$_{\pm1.0}$	$\underline{89.46}_{\pm1.39}$	88.64$_{\pm1.40}$
	MSA (ours)	-	-	**87.03**$_{\pm0.9}$	**75.03**$_{\pm1.6}$	**90.55**$_{\pm0.73}$	**91.34**$_{\pm0.85}$

[1] Cross-dataset, within-species (human-to-human) and within-modal transfer: Train on human sEEG data from NICU or CHSZ dataset, and test on another human sEEG dataset. There are no channel differences between NICU and CHSZ datasets, thus the proposed ResizeNet+MSA approach was not performed in this setting.

[2] Cross-dataset, within-species (human-to-human) and cross-modal transfer: Train on human iEEG/sEEG dataset, and test on human dataset with another modal, i.e., iEEG to sEEG, or sEEG to iEEG. Input discrepancies exist in this setting.

[3] Cross-dataset, multi-species [(canine+human)-to-human] and multi-modal transfer: Canine iEEG and human iEEG/sEEG datasets were combined as the training set, and CHSZ was used as the test dataset. This setting used two species and two modal data to train the models, so that input discrepancies exist.

[4] Baseline: Without using the proposed ResizeNet, the number of channels for both species was unified by eliminating the mismatching ones.

[5] ResizeNet+: Utilize the proposed ResizeNet projection to unify the number of channels for different modalities.

[6] Source Only: Train the model on all labeled data from the other dataset without employing any alignment strategy.

S6: Seizure prediction performance

Accurate forecasting of epileptic seizures greatly improves clinical epilepsy diagnosis and patient care. However, significant interspecies differences pose challenges for seizure prediction tasks. To evaluate the effectiveness of the proposed ResizeNet+MSA approach, extensive experiments were conducted for cross-species seizure prediction.

The Kaggle seizure prediction dataset from the seizure forecasting competition [19] was used, featuring epilepsy recordings from five canines and two humans undergoing extended iEEG monitoring. The data consist of 10-minute interictal and preictal clips. Substantial differences between canine and human recordings include sampling rates (400 Hz for canines, and 5000 Hz for humans) and electrode configurations (15 or 16 subdural electrodes for canines, and 15 or 24 intracranial channels for humans), reflecting cross-species characteristics similar to those in the seizure detection task. Additionally, the dataset exhibits a significant class imbalance between interictal and preictal trials. To address this imbalance, a subset of interictal samples was selected. For consistency with the seizure detection task, human iEEG signals were downsampled to 400 Hz, and each 10-minute clip was segmented into 10-second segments. Detailed characteristics of the dataset are summarized in Table S7.

Semi-supervised Canine-to-Human transfer experiments, with different portions of labeled trials, were conducted to evaluate three approaches: Within, Comb., and the proposed ResizeNet+MSA. Both Comb. and ResizeNet+MSA employed EA as a preprocessing step. The results, presented in Table S8, demonstrate that:

1. Comb. achieved superior performance compared to Within with an improvement of 6%, highlighting the benefits of incorporating data from another species.
2. The proposed ResizeNet+MSA achieved an additional 4.6% improvement over Comb., demonstrating its effectiveness in mitigating cross-species discrepancies and enabling better alignment across species.

Table S7. Characteristics of the five canine subjects and two human subjects in the Kaggle Prediction dataset.

Species	ID	# Channels	Sampling rate (Hz)	# Seizures	# Preictal trials	# Interictal trials	# Total trials
Canine	1	16	400	22	1440	10,000	11,440
	2	16	400	47	2520	10,000	12,520
	3	16	400	104	4320	10,000	14,320
	4	16	400	29	5820	10,000	15,820
	5	15	400	19	1800	10,000	1,1800
Human	1	15	5,000	5	1,080	3,000	4,080
	2	24	5,000	41	1,080	2,520	3,600

Table S8. Average semi-supervised cross-species seizure prediction transfer AUC (%) in Canine-to-Human seizure prediction. The best average performance of each task is marked in bold.

Approach	Portion of labeled trials				
	5%	10%	15%	20%	Avg.
Within	$62.82_{\pm 0.9}$	$72.81_{\pm 0.7}$	$78.24_{\pm 2.2}$	$82.61_{\pm 1.6}$	74.12
Comb.	$76.62_{\pm 1.0}$	$82.11_{\pm 0.4}$	$83.80_{\pm 1.4}$	$84.59_{\pm 1.8}$	81.78
ResizeNet+MSA	$\mathbf{81.02}_{\pm 1.9}$	$\mathbf{87.46}_{\pm 1.0}$	$\mathbf{87.68}_{\pm 1.2}$	$\mathbf{89.58}_{\pm 1.6}$	**86.44**

REFERENCES

1. He H and Wu D. Transfer learning for brain-computer interfaces: A Euclidean space data alignment approach. *IEEE Trans. on Biomed. Eng.* 2020; **67**: 399–410.
2. Long M, Zhu H, Wang J *et al.* Deep transfer learning with joint adaptation networks. In: *Proc. Int'l Conf. Mach. Learn.*, Sydney, Australia, 2208–17, Aug. 2017.
3. Zhu Y, Zhuang F, Wang J *et al.* Deep subdomain adaptation network for image classification. *IEEE Trans. on Neural Netw. Learn. Syst.* 2020; **32**: 1713–22.
4. Hinton G, Vinyals O and Dean J. Distilling the knowledge in a neural network. In: *Proc. Adv. Neural Inf. Process. Syst.*, Montréal, Canada, 1–9, Dec. 2015.

5. Long M, Cao Y, Wang J *et al.* Learning transferable features with deep adaptation networks. In: *Proc. Int'l Conf. Mach. Learn.*, Lille, France, 97–105, Jul. 2015.
6. Gretton A, Borgwardt KM, Rasch MJ *et al.* A kernel two-sample test. *J. Mach. Learn. Res.* 2012; **13**: 723–73.
7. Jin Y, Wang X, Long M *et al.* Minimum class confusion for versatile domain adaptation. In: *Proc. European Conf. Comput. Vis.*, Glasgow, UK, 464–80, Aug. 2020.
8. Liang J, Hu D, Wang Y *et al.* Source data-absent unsupervised domain adaptation through hypothesis transfer and labeling transfer. *IEEE Trans. Pattern Anal. Mach. Intell.* 2022; **44**: 8602–17.
9. Zagoruyko S and Komodakis N. Paying more attention to attention: Improving the performance of convolutional neural networks via attention transfer. In: *Proc. Int'l Conf. Learn. Represent.*, Toulon, France, 1–13, Apr. 2017.
10. Huang Z and Wang N. Like what you like: Knowledge distill via neuron selectivity transfer. ArXiv:1707.01219, 2017.
11. Tung F and Mori G. Similarity-preserving knowledge distillation. In: *Proc. IEEE/CVF Int'l Conf. on Comput. Vis.*, Seoul, Korea, 1365–74, Oct. 2019.
12. Park W, Kim D, Lu Y *et al.* Relational knowledge distillation. In: *Proc. IEEE/CVF Conf. Comput. Vis. Pattern Recognit.*, Long Beach, CA, USA, 3967–76, June 2019.
13. Passalis N and Tefas A. Learning deep representations with probabilistic knowledge transfer. In: *Proc. European Conf. on Comput. Vis.*, Munich, Germany, 268–84, Sep. 2018.
14. Peng B, Jin X, Liu J *et al.* Correlation congruence for knowledge distillation. In: *Proc. IEEE/CVF Int'l Conf. on Comput. Vis.*, Seoul, Korea, 5007–16, Oct. 2019.
15. Baldassano SN, Brinkmann BH, Ung H *et al.* Crowdsourcing seizure detection: Algorithm development and validation on human implanted device recordings. *Brain* 2017; **140**: 1680–91.
16. Ihle M, Feldwisch-Drentrup H, Teixeira CA *et al.* EPILEPSIAE–A European epilepsy database. *Comput. Methods Programs Biomed.* 2012; **106**: 127–38.
17. Wang Z, Zhang W, Li S *et al.* Unsupervised domain adaptation for cross-patient seizure classification. *J. Neural Eng.* 2023; **20**: 066002.
18. Stevenson NJ, Tapani K, Lauronen L *et al.* A dataset of neonatal EEG recordings with seizure annotations. *Sci. Data* 2019; **6**: 1–8.
19. Brinkmann BH, Wagenaar J, Abbot D *et al.* Crowdsourcing reproducible seizure forecasting in human and canine epilepsy. *Brain* 2016; **139**: 1713–22.
